# Palaearctic flea beetle *Phyllotretaochripes* (Curtis) (Coleoptera, Chrysomelidae, Galerucinae), herbivore of *Alliariapetiolata* (garlic mustard), new to North America

**DOI:** 10.3897/BDJ.12.e135576

**Published:** 2024-12-12

**Authors:** Hume B Douglas, George Hammond, Tyler W Smith, Jessie Mutz, Alexander S Konstantinov

**Affiliations:** 1 Agriculture and Agri-Food Canada, Ottawa, Canada Agriculture and Agri-Food Canada Ottawa Canada; 2 City of Ann Arbor Natural Area Preservation, Ann Arbor, United States of America City of Ann Arbor Natural Area Preservation Ann Arbor United States of America; 3 Department of Ecology & Evolutionary Biology, University of Tennessee, Knoxville, United States of America Department of Ecology & Evolutionary Biology, University of Tennessee Knoxville United States of America; 4 Systematic Entomology Laboratory, MRC-168 Washington, United States of America Systematic Entomology Laboratory MRC-168 Washington United States of America

**Keywords:** invasive alien species, adventive species, biological control, garlic mustard, *Alliariapetiolata*, weed biology, woodland

## Abstract

**Background:**

The univoltine leaf beetle *Phyllotretaochripes* (Curtis, 1837b) is native to the Palaearctic Region from Japan to western Europe.

This species was previously evaluated as a potential biological control agent against invasive populations of the woodland weed *Alliariapetiolata* (Bieb.) Cavara & Grande (Brassicaceae) in North America, but rejected because it could harm native and at-risk populations of Brassicaceae.

**New information:**

First North American records are presented for *Phyllotretaochripes* (Curtis, 1837). Specimens were examined from the USA: Illinois, Maryland, Michigan, Ohio and Pennsylvania. Internet photographs of apparent additional individuals from USA: Indiana, Michigan, Minnesota, Ohio, Pennsylvania, Tennessee, Wisconsin and Canada: Ontario were also examined. DNA barcoding analysis showed high genetic variability and possible cryptic species within European populations of *P.ochripes*. Diagnostic information is presented to distinguish *P.ochripes*. from other North American Chrysomelidae and a species distribution model to assess its potential spread in North America is presented.

*Phyllotretaochripes* breeds on invasive garlic mustard, *Alliariapetiolata* (Bieb.) Cavara & Grande (Brassicaceae) and also non-native *Rorippaamphibia* (L.) Besser and other species of Brassicaceae.

A species distribution model and the range of its host plant *A.petiolata*, indicates the most suitable conditions for this species are in humid areas of eastern North America. However, most of the known records of this species were discovered in areas projected to have low suitability. This is likely a consequence of sampling bias towards western Europe and away from the eastern Asian portion of its native range. The United States of America and Canada are now known to be home to 72 or more species of adventive Chrysomelidae.

## Introduction

Leaf beetles are drivers of ecological change in terrestrial biomes, acting as plant pests, biological control agents and often as abundant herbivores (Myers and Sarfraz 2017). Recent studies have documented several newly-recorded species of European Chrysomelidae in cool-temperate parts of eastern North America (Deczynski 2019, Douglas et al. 2021b, Klimaszewski et al. 2020, Pentinsaari et al. 2019, Douglas et al. 2023).

Garlic mustard, *Alliariapetiolata* (M.Bieb.) Cavara & Grande is a member of Brassicaceae, is native to the Palaearctic Region and has become abundantly established in many deciduous forests in eastern North America (Nuzzo 1993). This species displaces native understory plants, mainly through allelopathic inhibition of germination (Prati and Bossdorf 2004) and disruption of mycorrhizal associations (Roche et al. 2020). It reduces populations of native herbivorous insects by acting as a non-productive oviposition trap (Augustine and Kingsolver 2018). Stinson et al. (2018) found garlic mustard to further modify forest understory plant communities by promoting population growth of litter-consuming, non-native earthworms. This highly competitive weed was a focus of efforts to identify potential biological control agents from its native range because herbivores were causing little damage to this plant in North America. Efforts to identify biological control agents against invasive *A.petiolata* in North America began after 1990. *Phyllotretaochripes* (Curtis, 1837) was evaluated and rejected as a possible agent because it could complete development on other valued species of Brassicaceae (CABI 2023, Verdyck 2008). Since then, Dr. Robertson Davenport (Natural Area Preservation Unit, City of Ann Arbor, Michigan, USA) noticed substantial insect feeding damage on *A.petiolata* in a city nature preserve. He knew that such damage was unexpected and alerted city biologists about the beetle causing the damage.

Here, we report discoveries of a new adventive Chrysomelidae in USA and Canada by non-entomologist biologists. *Phyllotretaochripes* is native throughout Europe, Iran and in north-eastern Asia (Doberl 2010). This species is known to mainly develop on *A.petiolata* in Europe (Rheinheimer and Hassler 2018) and we discuss the ecology of *P.ochripes* mainly in the context of that host plant. We also discuss our findings in the context of potential benefits in reducing invasive garlic mustard populations and potential harms to native Nearctic Brassicaceae species.

## Materials and methods

In spring 2017, R. Davenport found leaf beetles feeding on garlic mustard in Ann Arbor (Michigan, USA). He alerted city staff and requested identification from the United States Department of Agriculture’s Animal and Plant Health Inspection Service (APHIS). APHIS Plant Protection Officer, Elizabeth Pentico, identified the specimens as *P.ochripes*, noting that the species was not known to occur in North America. GH also tentatively identified the specimens as *P.ochripes* based on morphology and presence on *Alliaria*. GH also found several prior online iNaturalist observations of *P.ochripes* from USA, but no records in peer-reviewed scientific journals. Ann Arbor City Natural Areas Protection staff contacted taxonomic specialists and searched for additional specimens, finding them on garlic mustard at several city sites. They photographed and collected beetles from host plants and sent them to HD and AK for examination.

HD compared dissected specimens to North American (Smith 1985) and European (Mohr 1966, Doguet 1994) taxonomic literature, expert managed internet websites with identified images of beetles (Borowiec 2011) and identified specimens from the Canadian National Collection of Insects, Arachnids and Nematodes (CNC, Ottawa, Canada). AK compared the Michigan, Maryland and Tennessee specimens to *Phyllotreta* specimens preserved at the United States National Museum (USNM, Washington, USA). Diagnoses by Mohr (1966) and Doguet (1994) for *P.ochripes* were tested for applicability in North America by comparison to the Smith (1985) revision and key to North American *Phyllotreta* species with pale elytral markings. Specimens collected in North America and European specimens at CNC and USNM were compared to the Smith's revision and to European diagnoses by Mohr (1966) and Doguet (1994) to generate a diagnosis for *P.ochripes* in North America.

To search for additional evidence about the possible distribution of *P.ochripes* in USA and Canada, HD reviewed over 700 iNaturalist observations (iNaturalist contributors and iNaturalist 2024) attributed to genus *Phyllotreta* in a rectangular area bounded approximately by Gander, Newfoundland in the northeast, Jacksonville, Florida in the southeast and Austin, Texas in the southwest. This defined area was meant to capture the populated cool-temperate areas of eastern North America and also most of the range of host plant *Alliariapetiolata* in North America. All *Phyllotreta* specimens with pale elytral markings and either occurring on *Alliaria* or having pale profemora or mesofemora were considered potential *P.ochripes* specimens. The elytral markings of photographed individuals were then compared to *P.ochripes* (looking for apical pale expansion covering most of elytra, but not reaching suture or margin) to confirm online records. HD added online identifications to iNaturalist records that also agreed with the diagnostic characters below as identified as probable *P.ochripes* occurrences.

For DNA analysis, we sent a single leg from three specimens to the Centre for Biodiversity Genomics (CBG, University of Guelph, Guelph, Ontario, Canada). There, the legs were each placed in a well in a 96-well microplate prefilled with 10 µl of 96% ethanol. Each specimen was also photographed and the resulting image was uploaded to the Barcode of Life Database (BOLD; Ratnasingham and Hebert (2013)) along with the label data. The DNA extraction, polymerase chain reaction amplification and Sanger sequencing of the cytochrome oxidase subunit 1 barcode region were performed for all specimens at the CBG, using standard protocols as outlined by Pentinsaari et al. (2019). Primers *C_LepFoIF* and *C_LepFoIR* (Woodcock et al. 2013) were used for polymerase chain reaction amplification. Sequences were obtained through unidirectional analysis. Details on the polymerase chain reaction and sequencing protocols for each specimen are provided in the public BOLD dataset information below.

Detailed collection information for each specimen, including both DNA-barcoded material and other specimen records, as well as GenBank accession numbers for the barcode sequences, are provided in the Taxon Treatment section. All sequences, details on polymerase chain reaction and sequencing primers, photographs and full collection data for the DNA-barcoded specimens are available through a public dataset on BOLD (https://doi.org/10.5883/DS-PHYLOCHR). Specimen occurrences were mapped using SimpleMappr (Shorthouse 2010).

As a preliminary assessment of the potential distribution of *P.ochripes* in North America, TWS prepared a species distribution model using the programme Maxent version 3.4.4 (Phillips et al. 2017). All records from Europe and Asia were downloaded from GBIF.org (2024a) using the rgbif R package (Chamberlain et al. 2024). Records were filtered to remove duplicates and records with coordinates that coincided with museums using the CoordinateCleaner R package (Zizka et al. 2019). Records were thinned to one observation per 10 minute grid cell to reduce spatial clustering. Following filtering, 631 observations were retained for model training. We confirmed that the GBIF records agreed with reports in published databases (Cox 2007, Clayhills et al. 2024). However, while the GBIF dataset included records from France and the United Kingdom in the west to Kazakhstan in the east, Doberl (2010) indicates that it has also been documented across Russia to Japan. Our modelling work was thus limited to records available from Europe and Western Asia because we could obtain best modelling results using only precise georeferenced records from GBIF.

We obtained climate rasters from Climond (Kriticos et al. 2011) at 10' resolution (approximately 19 km^2^ at the Equator) and selected the eight variables recommended by Petitpierre et al. (2017) for optimising model transferability at continental scale (BIO1: mean annual temperature, BIO4: temperature seasonality, BIO11: mean temperature of coldest quarter, BIO10: mean temperature of warmest quarter, BIO15: precipitation seasonality, BIO16: precipitation of wettest quarter, BIO28: annual mean moisture index, BIO31: moisture index seasonality). This suite of variables is assumed to reflect the physiological limits of most taxa. While it would be preferable to select variables based on experimentally validated physiologically thresholds, such data are not available for *P.ochripes*.

To address spatial bias in occurrence records, we used a bias grid to select background points for model training. This approach estimates the sampling effort used to locate occurrence records with a larger set of records that are similar in detectability and research interest (Phillips et al. 2009, Syfert et al. 2013). We selected tribe Alticini (which includes *Phyllotreta*) as the target group, as many of the other Chrysomelidae are larger and more colourful and consequently likely to be collected at a higher rate.

We included all GBIF records (GBIF.org 2024b) for Alticini genera listed in Doberl (2010), with the same filtering as described above for *P.ochripes*, which resulted in a cleaned dataset of 112,266 records. We used these records to calculate a Gaussian Kernel Density estimate (GKD) using the *density* function of the spatstat R package (Baddeley et al. 2015) with the default settings. We then used the GKD values as weights in randomly selecting 10,000 background points to include in model training.

We constructed Maxent models including all combinations of linear, quadratic and hinge features. We excluded threshold and product features, as these have been shown to increase computation time, complicate model interpretation, while adding only very minimally to model performance (Phillips et al. 2017). We varied the regularisation multiplier from 1 to 3, in order to identify the optimal trade-off between model over-fitting and underfitting (i.e. too many or too few features), as recommended by Warren and Seifert (2011) and implemented in the ENMeval R package (Kass et al. 2021). The default Maxent CLOGLOG output, which ranges from 0-1, was used to interpret projections, with values above the median (i.e. the 50 percentile) considered to be highly suitable, values between the 5 and 50 percentile as moderately suitable and values between the 1 and 5 percentile as low suitability. As an aid in visual interpretation of the results through mapping, we obtained all GBIF records for the host plant *Alliariapetiolata* (GBIF.org 2024c). All analyses were conducted in R version 4.4.0 (R Core Team 2024).

## Data resources

The new specimen occurrence data reported in this paper are deposited at GBIF, the Global Biodiversity Information Facility: https://doi.org/10.15468/hutmv7. Online photographic records discussed in the article are available at GBIF, iNaturalist user records: https://doi.org/10.15468/dl.t9cqzg.

## Taxon treatments

### Phyllotreta ochripes

(Curtis, 1837)

0E049EEA-8C47-5B5C-ABBD-E095B2CF787F

https://www.gbif.org/species/4462267

#### Diagnosis

*Phyllotretaochripes* can be recognised in North America and Europe by the following characteristics adapted from Mohr (1966) and Doguet (1994): body 2.0-2.4 mm; body black with pale stripe or two spots on each elytron; antennae with antennomeres I-III pale in male, with antennomere V two times longer than antennomere VI and somewhat wider; female antennae with antennomeres I-VI pale, antennomere V 1.8 times longer than VI and cylindrical; pale elytral stripes with basal-mesal, sub-basal lateral and apical expansions, pale area occupying most of apical ¼, but not reaching suture or apex; most with legs pale, except femora of hind legs. Here, no other North American *Phyllotreta* with pale elytral markings has all of the anterior- and mid-legs all pale.

#### Distribution

We examined 24 specimens of P.ochripes from USA: Illinois, Maryland Michigan, Ohio and Pennsylvania. Specimen data are available through GBIF.org in Douglas (2024). Additional individuals were seen in 29 online iNaturalist citizen-science observations from USA: Indiana, Michigan, Minnesota, Ohio, Pennsylvania, Wisconsin and Canada: Ontario (iNaturalist contributors and iNaturalist 2024). These photos were each identified by iNaturalist contributors as being of *P.ochripes* and most were confirmed by HD to match *P.ochripes* and no other North American chrysomelid species. In addition to having pale fore- and mid-legs and elytral markings consistent with only *P.ochripes*, the distribution of online records was also broadly concordant with that of vouchered specimens (Fig. 3). Additionally, 11 of 29 online records matching *P.ochripes* were photographed from plants either identified as *A.petiolata* by the photographers or visually matching *A.petiolata*. Two additional plants were photographed on plants appearing possibly consistent with *A.petiolata*, two were photographed on other plant species and the remainder were photographed on non-plant surfaces. Overall, there are strong collections and online evidence of *P.ochripes* across much of north-eastern USA and in southern Ontario.

We present specimen and photographic evidence of 53 individuals from multiple sites in USA: Maryland, Michigan, Minnesota, Ohio, Pennsylvania, Tennessee, Wisconsin and Canada: Ontario, separated by over 1300 km over six years (earliest observation: 2017, Fig. 2). Photographs on another online citizen-science platform show evidence that this species was already present in USA (Pennsylvania and Tennessee) in 2014 (Moorman 2014, Rossenfeld 2014). These led us to conclude that *P.ochripes* is established at multiple sites in North America.

#### Biology

*Phyllotretaochripes* has been observed here to make holes fully through all tissue layers of leaves. This is unlike the weevil biological control agent against *A.petiolata*. Here, *Ceutorhyncusscrobicollis* Nerensheimer & Wagner (Coleoptera, Curculionidae) causes similar-sized window-pane type damage, where a transparent cuticular layer remains over the damaged area (CABI 2018). *Phyllotretaochripes* has been demonstrated to have attraction to allyl isothiocyanate (Tóth et al. 2007), the chemical responsible for the spicy taste of some Brassicaceae.

#### Notes

The external morphology and genitalia of specimens from USA best matched species concepts of *P.ochripes*, aligning with European specimens at the CNCI and USNM (Fig. 1). However, the aedeagus of North American specimens had smaller apical concavities than some European specimens. Specimens were all hand-collected from forest understory *A.petiolata* plants with leaf holes present.

##### DNA Barcoding Results

Analysis of the DNA-barcoded USA specimens of *Phyllotreta* through the BOLD Identification Engine resulted in an at least 99.5% match with some of the 348 publicly available *P.ochripes* sequences. This specimen shares a BOLD Barcode Index Number (Ratnasingham and Hebert 2013) with two *P.ochripes* specimens from the country of Georgia for which public data were not available (BOLD:AEH0075), indicating a maximum p-distance of 0.73%. However, this bin had a larger p-distance of 3.95% from a second bin, corresponding to *P.ochripes* from western Europe. The morphological identification of these specimens as best matching *P.ochripes* (although with some aedeagal differences), including their presence on *Alliaria* and the finding that our DNA barcoded specimen closely matched DNA from eastern European specimens, all support the conclusion that the North American specimens are *P.ochripes*. However, the finding that BOLD has identified a 4% COI sequence divergence within European *P.ochripes* may be important. This suggests that more research is needed to determine whether an additional cryptic species is contained within the current concept of *P.ochripes*.

## Analysis

Two species distribution models were identified as optimal: regularisation 1, linear, quadratic and hinge features; and regularisation 1, hinge features only. We selected the latter model as it was simpler (i.e. only one feature class). This model had an average continuous Boyce index of 0.968 and AUC of 0.884 in cross-validation tests, indicating good performance. The top three variables were mean temperature of warmest quarter (34.5% contribution), precipitation seasonality (31.9%) and temperature seasonality (20.1%). The response curves for these variables suggest the optimal conditions for *P.ochripes* include summer temperature range between 10° and 24°C and precipitation seasonality less than 15.8%.

The model predicts the highest suitability in north-western Europe and the southern United Kingdom, declining eastwards (Fig. 4). Notably, the distribution of *Alliariapetiolata* extends further into southern Europe than either the distribution records for *P.ochripes* or the extent of its predicted habitat suitability. This suggests the distribution of *P.ochripes* does not simply mirror that of its host plant, but is restricted by additional factors as well. The same pattern is evident at the eastern margin of the documented distribution of *P.ochripes* in Russia and Kazakhstan. However, as we know the beetle's range extends well beyond this region (according to Doberl (2010)), both the records and the model projections in eastern Europe and western Asia must be considered provisional.

Projecting this model to North America, the most suitable areas are shown in relatively humid locations along the east coast (Nova Scotia and eastern Newfoundland) and in the Appalachian Mountains, particularly in the southern Blue Ridge area along the North Carolina/Tennessee border (Fig. 5). Notably, most of the records for *P.ochripes* are in the low suitability regions or entirely outside of the Maxent projections (e.g. in Minnesota, Wisconsin and Illinois). The records for *P.ochripes* fall in the western portion of the current range of *Alliariapetiolata*; the plant is also common further eastwards into Nova Scotia and with scattered records across further west in USA.

We did not include other secondary host plants in our analysis. However, *P.ochripes* is known to feed on *Rorippa* spp., including *R.palustris* (Blossey et al. 2003). This species is found in every state, province and territory in Canada and the USA (NatureServe 2024).

## Discussion

### Adventive species biology

A former pathway for European beetles with root feeding larvae to arrive in North America was with ornamental plants imported to Canada and USA from 1960 to 1965. This plant material is known to have contained beetle larvae, including those of several species discovered as established adventive since 2000 (Douglas 2011, Douglas et al. 2021a). The timing of the discovery of *P.ochripes* in North America suggest that the same pathway of introduction is possible here. That larval *P.ochripes* feed on roots of *A.petiolata*, an abundant plant in many habitats (including plant nurseries), suggests that *P.ochripes* was perhaps also introduced with European horticultural plants with soil. While we know of no larvae of *P.ochripes* intercepted in imported plant material, these would have been small and difficult to detect. However, the larger larvae of some European Elateridae were detected in such shipments and some of these species were later found as adventive in North America (Douglas 2011).

*Phyllotretaochripes* is known to inhabit humid forests, meadows and shorelines using *Alliaria* and other Brassicaceae as host plants. In its native range, *P.ochripes* also feeds on other Brassicaceae including watercress (*Nasturtiumofficinale* R. BR.), rapeseed or canola (*Brassicanapus* L.), woad (*Isatis* spp.), wall rocket (*Diplotaxis* spp.) and various species of *Cardamine* L. (summarised in Rheinheimer and Hassler (2018)), indicating that it is likely to also feed on economically important Brassicaceae in the Nearctic, as well as on native species (possibly including the federally endangered species *Cardaminemicranthera* Rollins (US Fish and Wildlife Service 2023)). We do not know which of these other plant genera can support larval development. Here, the arrival of *P.ochripes* in North America is likely to cause both beneficial and harmful effects on plant communities. This additional North American record indicates that Canada and USA together host 72 to 82 species of adventive Chrysomelidae (Douglas 2024, Douglas et al. 2021a). This is the fourth or fifth species of *Phyllotreta* introduced into North America (Douglas et al. 2021a). We anticipate publication of additional new records of European Chrysomelidae in North America in the near future.

*Phyllotretaochripes* represents the third adventive insect found on *A.petiolata* after two Palaearctic aphid species were also found feeding on garlic mustard in USA (Lagos-Kutz et al. 2022). Additionally, the highly-damaging stem and root crown mining weevil, *Ceutorhynchusscrobicollis*, has been released in Ontario as an approved biological control agent against *Alliariapetiolata* (McTavish et al. 2024), thereby adding a major herbivore in part of the introduced range of garlic mustard. Together, introduced species are likely to increase herbivore pressure on this plant species and reduce its invasiveness in North America.

### Conclusions

*Phyllotretaochripes* has been established in North America in Canada: Ontario and USA: Indiana, Maryland, Michigan, Minnesota, Ohio, Pennsylvania, Tennessee and Wisconsin on introduced *Alliariapetiolata* (Bieb.) Cavara & Grande (Brassicaceae) plants. Numbers of recorded adventive Chrysomelidae for Canada and America, north of Mexico are updated to reflect this finding.

## Supplementary Material

XML Treatment for Phyllotreta ochripes

## Figures and Tables

**Figure 1.**
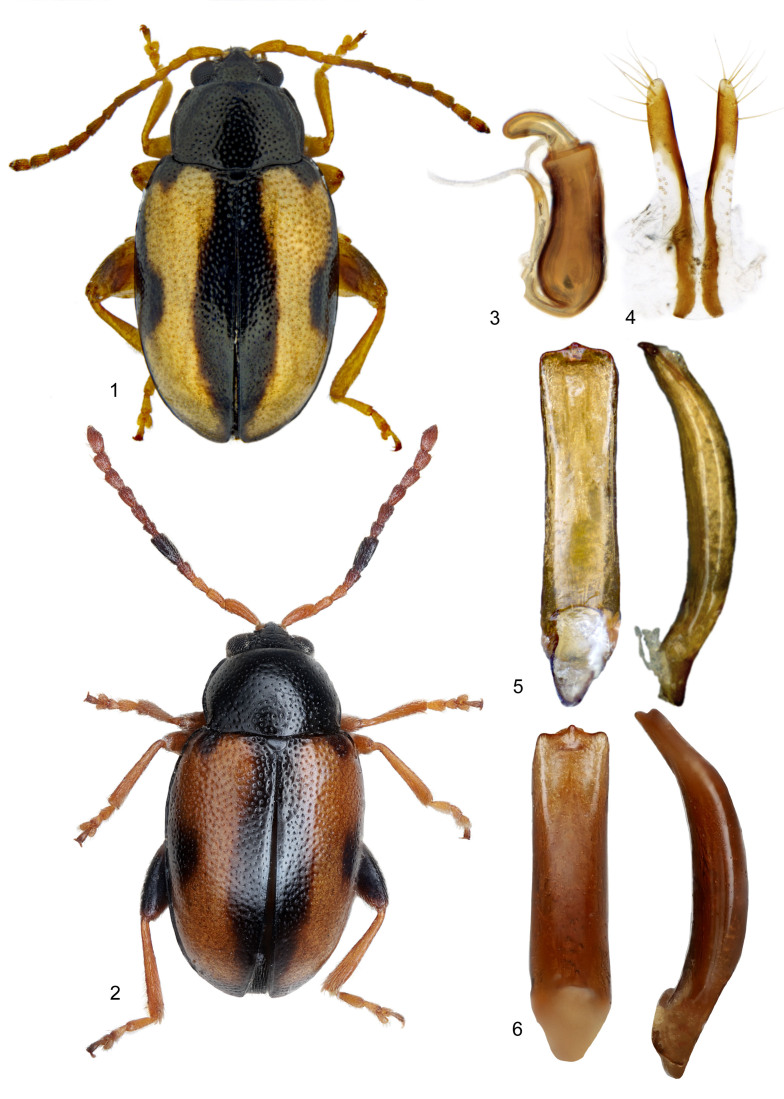
Morphology of *Phyllotretaochripes*. **1** Male from Michigan USA, dorsal habitus; **2** Male from Hungary, dorsal habitus; **3** Female from Michigan, USA, spermatheca; **4** Female from Michigan, USA, vaginal palpi; **5** Male from Michigan, USA, aedeagus; **6** Male from Hungary, aedeagus. Illustrations: K Savard (AAFC).

**Figure 2a.**
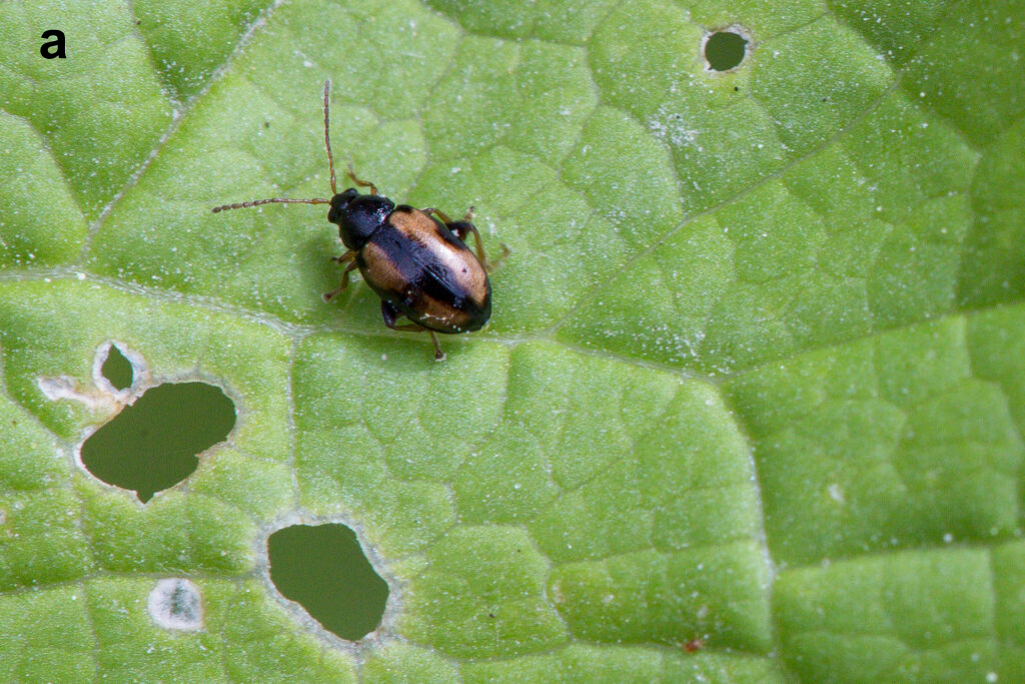


**Figure 2b.**
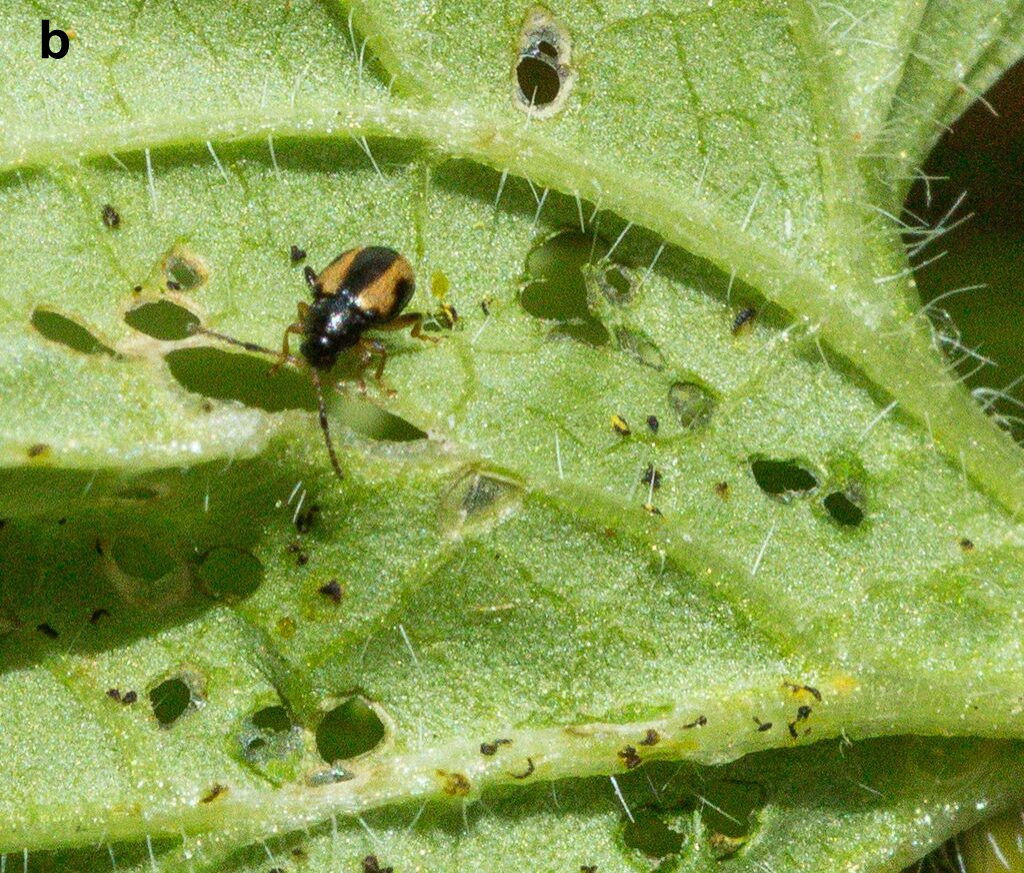


**Figure 3.**
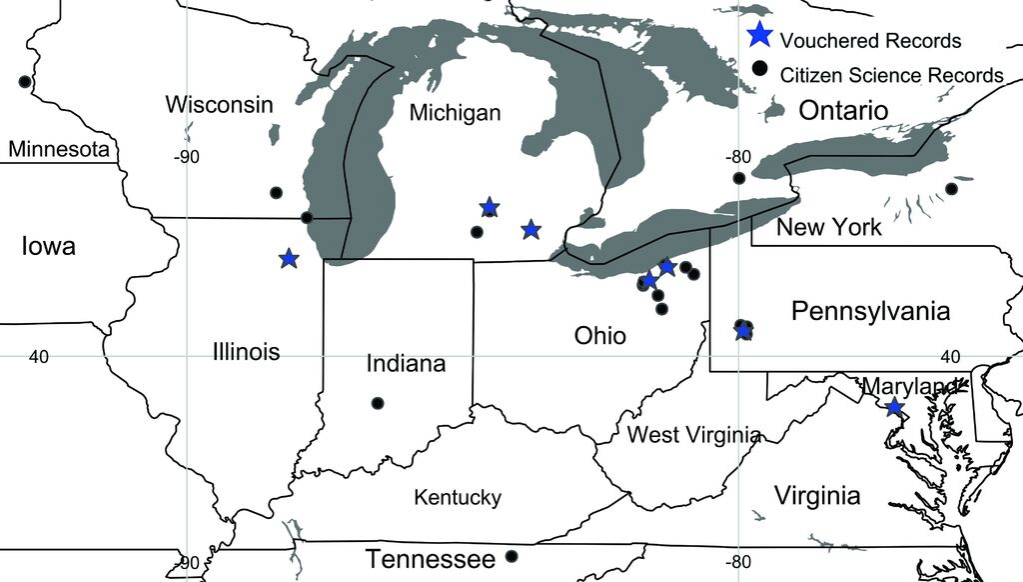
Map of vouchered records (blue star) and citizen-science records (black circle) of *Phyllotretaochripes* from USA and Canada.

**Figure 4.**
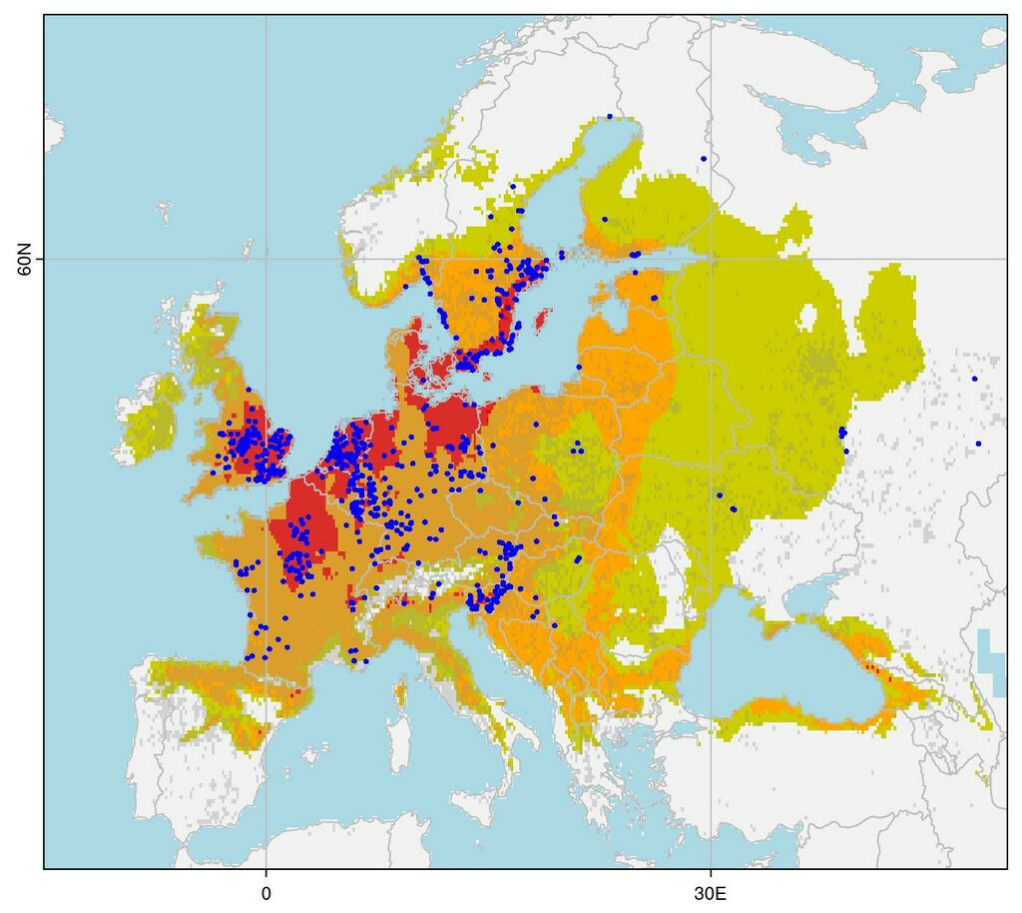
Distribution of *Phyllotretaochripes* in its native range. Blue points show GBIF records. Colours indicate Maxent suitability models: dark/red areas are the highest suitability (50 percentile and above, CLOGLOG > 0.73), medium/orange areas are moderate suitability (5 percentile, 0.20 < CLOGLOG < 0.73) and light/olive areas are low suitability (1 percentile, 0.05 < CLOGLOG < 0.20). Shading indicates GBIF records for the host plant *Alliariapetiolata*.

**Figure 5.**
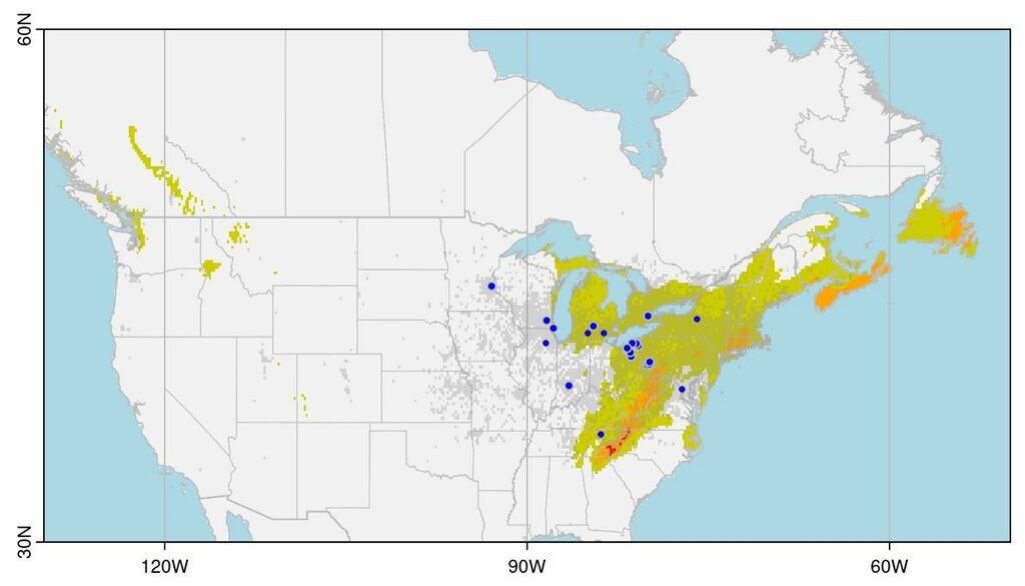
Maxent suitability map for *Phyllotretaochripes* in North America. Blue points show known occurrences. Colouring indicates Maxent suitability values: dark/red areas are the highest suitability, medium/orange areas are moderate suitability and pale/olive areas are low suitability. Grey shading indicates GBIF records for the host plant *Alliariapetiolata*.
